# Pre-pandemic socio-economic status and changes in employment during the first lockdown (2020) on the health of middle-aged people in England: a longitudinal study

**DOI:** 10.1186/s12889-022-14248-9

**Published:** 2022-10-12

**Authors:** Stefania D’Angelo, Georgia Ntani, Ilse Bloom, Karen Walker-Bone

**Affiliations:** 1 MRC Lifecourse Epidemiology Centre, University of Southampton, Southampton, UK; 2 MRC Versus Arthritis Centre for Musculoskeletal Health and Work, MRC Lifecourse Epidemiology Centre, University of Southampton, Southampton, UK; 3 Monash Centre for Occupational and Environmental Health, Monash University, Melbourne, Australia

**Keywords:** COVID-19, older workers, employment changes, socio-economic status, self-rated health, mental health, physical health

## Abstract

**Background:**

The COVID-19 pandemic markedly disrupted people’s lives. It caused higher mortality and morbidity amongst individuals from poorer socio-economic position (SEP). It is well-recognised that job loss has a negative impact on health. We hypothesised that health effects of the pandemic on middle-aged people might be different depending on SEP and changes in employment.

**Methods:**

Data are from the Health and Employment After Fifty (HEAF), a cohort recruited 2013–2014 when aged 50–64 through 24 English general practices. At baseline and annually since, participants completed a questionnaire reporting about demographics, employment, health, lifestyle, and finances. In 2021 we sent an e-survey to all contactable HEAF participants, asking about effects of the first lockdown (March-July 2020). Outcomes were participants’ perception of worsening of mental, physical health, and self-rated health (SRH) since lockdown. Associations between SEP, COVID-19 related employment changes and health were explored with Poisson regression with robust standard error, with adjustment for age, sex, and pre-pandemic SRH.

**Results:**

In total, 2,469 (53%) returned a usable questionnaire, amongst whom 2,344 provided complete information for these analyses (44% men, mean age 65.7 years). Worsening of mental, physical or SRH since lockdown was reported by 21%, 27% and 17% respectively. Mutually adjusted models showed that reporting struggling financially pre-pandemic (versus living comfortably) was associated with an increased risk of deterioration in: mental (RR = 2.0, 95%CI 1.7–2.5), physical health (RR = 2.0, 95%CI 1.6–2.3), and SRH (RR = 1.6, 95%CI 1.2–2.1). Participants working from home during lockdown and those who lost their job (as opposed to those with unchanged employment) were at increased risk of reporting deterioration in mental health and SRH.

**Conclusion:**

In a cohort of older workers, working from home, job loss and poorer pre-pandemic SEP were all associated with worsening of mental health and SRH since lockdown.

## Background

On the 23rd of March 2020 the UK entered its first national lockdown, a measure taken by the government, aiming to limit the spread of COVID-19 and release pressure on the National Health Service (the UK’s publicly funded health service). The UK labour market was substantially impacted by the lockdown which required all non-essential businesses (e.g. hospitality and non-food retail) to close their doors. As a result, almost a quarter of all UK businesses were forced to temporarily close or pause trading as of April 2020. [1] Where possible, non-essential businesses were expected to continue to operate with their labour force working from home. Early in the pandemic, the UK government introduced two policies aimed at supporting businesses and those who were self-employed, the Coronavirus Job Retention Scheme (CJRS) and the Self-Employment Income Support Scheme (SEISS). The first scheme (which applied from 1 March 2020 and ended on 30 September 2021) provided grants to employers so they could retain and continue to pay staff during lockdown, by furloughing employees at up to 80% of their wages. [2] The SEISS (which applied from 30 April 2020 and ended on 30 September 2021) provided similar support to self-employed individuals who, if eligible, were able to receive a grant corresponding to 80% of their average monthly trading profits. [3] Data published by the Resolution Foundation show that approximately 15% of adults working in the pre-pandemic period were furloughed, while 3% lost their job and 4% saw their working hours and pay diminished. [4]

It is well known that unemployment and job loss are associated with poorer health [5, 6]. Given that employment status changed for many people, it is important to gather evidence on the effect of such changes on their health. Studies from the UK, US, and Australia have all reported associations between losing a job during lockdown and worsening of mental health, describing symptoms of depression, anxiety and feelings of loneliness. [7] [8] [9]. Being furloughed has also been found associated with an increased risk of developing depression and poor mental and/or physical health, compared with people who retained their employment. [9, 10] However, it is less clear what health effects there have been amongst those home working, with some studies finding no changes in mental health [11] and others finding an increase in back pain among those who started working from home [12]. Notably, much of the available data are from studies of younger people or the general working age population (aged 18 + years). However, in the UK, although furlough rates were greatest amongst younger workers (< 25 years) they were also very high amongst adults aged > 60 years. [13] This is important as older workers may be more likely to permanently exit the workforce once out of work [13, 14], and also because this age group are being targeted by government policies aimed at increasing the length of working lives to support the growing pension gaps in developed societies. Moreover, the age group 50 + deserves special attention as they are more likely to live with chronic disorders compared with younger age groups. [15] COVID-19 was seen to have a differential impact by socio-economic position (SEP) such that those from poorer SEP had higher mortality and morbidity[16].

Therefore, within this paper we aimed at investigating: (1) the effect of a change in employment during lockdown on mental, physical and self-rated health, among middle-aged people taking part in the Health and Employment After Fifty (HEAF) study; (2) the effect of SEP before the pandemic on the same health outcomes.

## Methods

This study used data from the HEAF COVID-19 survey as well as previously collected data from the wider HEAF study. HEAF is a cohort of people recruited 2013–2014 from 24 English GP surgeries when aged 50–64 years. Full details and methods of the study have been published previously [17]. Study participants completed a baseline questionnaire including information about their socio-demographic and lifestyle factors (which included smoking habits and body mass index among others), health, finances, employment status and working conditions (if they were in paid work). Those who consented were mailed follow-up questionnaires, approximately annually thereafter, gathering prospective information about changes that had occurred to their employment, lifestyle, finances, and markers of health. In 2020, during the first English lockdown, the HEAF study team obtained an ethics amendment to contact participants who had provided an e-mail address, with an online survey to enquire about the short-term consequences of the pandemic. The survey, which was sent out in February 2021, included 67 questions, was administered on the online platform Qualtrics (Provo, UT), and covered the period of the first lockdown (March - July 2020). Questions were asked about: personal experiences of COVID-19 and shielding, healthcare utilisation, employment circumstances before and after March 2020, finances, mental and physical health and social isolation, lifestyle factors and changes to people’s lives since the start of lockdown. Respondents were asked to re-confirm their consent for participation in this sub-study and were informed they could withdraw their consent at any time. COVID-19 related employment changes were derived by combining data about participants’ employment position before and during lockdown with categories being “already retired pre-lockdown”, “work unchanged”, “work from home”, “furloughed”, “no longer working”.

### Measures of SEP

SEP is commonly measured using information on either educational attainment, social class, income, or housing tenure. [18, 19] Actual income was not available in the HEAF study, but all participants had been asked annually about their perceptions of whether or not they were managing financially. All study participants were asked to describe their educational attainment at baseline by ticking the most appropriate of the following options: “No qualification/school only”; “Vocational training certificate”; “University degree or higher”. Social class was derived from self-reported occupation and industry amongst people in paid work at HEAF follow-up 4 (2017–2018), classified in accordance with the National Statistics Socio-economic classification (NS-SEC) [20]. Nine categories were used for analysis, namely “Managers, directors and senior officials”, “Professional occupations”, “Associate professional and technical occupations”, “Administrative and secretarial occupations”, “Skilled trades occupations”, “Caring, leisure and other service occupations”, “Sales and customer service occupations”, “Process, plant and machine operatives”, “Elementary occupations”. Housing tenure was self-reported at baseline as either “owned outright”, “owned with mortgage”, “rented” or “rented free”. Because of scarce data, “rented” and “rented free” were combined for the analyses (“living in rented accommodation”). Pre-pandemic self-perceived financial status was assessed at the latest pre-pandemic questionnaire (June 2019) with the following question: “How well are you managing financially?” with possible answers “living comfortably”, “doing alright”, “just about getting by”, “finding it difficult to make end meets”, “finding it very difficult to make end meets”. Because of sparse data in some categories, we recoded the variable as: “Living comfortably”, “Doing alright”, “Just about/struggling ”.

### Outcomes

The online questionnaire included the following two questions: Since the start of lockdown, how much do you agree with the statement: 1 “My mental health has deteriorated” and 2 “my physical health has deteriorated”. They were given five response options on a 5-Level Likert scale: “strongly agree”; “agree”; “neither agree nor disagree”; “disagree” and “strongly disagree”. For analyses, the five options were dichotomised so that “strongly agree” and “agree” were compared with the remaining three categories “does not agree”. Throughout the HEAF study, participants have been asked to rate their self-rated health (SRH) using the question: “in general, how would you say your health is?” with 5 options: “excellent”, “very good”, “good”, “fair” and “poor”. Within the COVID-19 e-survey, participants were asked this question twice (SRH in February 2020 and SRH during lockdown). For analysis, we compared the self-reported response of February 2020 with that reported during lockdown, assigning a score to any change in SRH between the two time points. This variable was then dichotomised as: “SRH worsened between the two time points” or “SRH remained the same or improved between the two time points”.

### Statistical analysis

The risk of any selection bias was tested by comparing socio-demographic characteristics and health status of participants who returned a usable questionnaire with those who did not using data from the HEAF baseline questionnaire (age, finances, educational attainment, home ownership, smoking status, obesity, and self-rated health). Characteristics of participants were reported, with numbers and percentages for categorical variables and means (SD) for normally distributed continuous outcomes. Prevalence of each health outcome was shown for the overall sample as well as for categories of predictors.

Since outcomes were common (prevalence > 10%), Poisson regression model with the option for robust standard error was used to explore associations between SEP variables, changes in employment and health outcomes, with results expressed as relative risks (RRs) and 95% Confidence Interval (95%CI). Estimates were adjusted for age, sex, and for pre-pandemic SRH (reported in 2019). We built separate models for each measure of SEP. Analyses were performed in Stata v17.0.

## Results

A total of 8,134 participants enrolled in the HEAF study at baseline. Of those, 5,454 remained consenting participants at the time of this survey amongst whom 4,665 had provided a valid email address and could therefore be invited to complete the online survey. In total, 2,469 (53% response rate) returned a usable questionnaire, but 39 people provided no information about their employment and 55 people were unemployed before and during lockdown and were excluded. We further limited the analyses to participants with non-missing data on at least one measure of SEP, response about employment change since lockdown and at least one outcome (n = 2,344). 40% of the sample was in work in February 2020 (just before the start of lockdown).

As reported in Table 1, participants who returned a usable COVID-19 questionnaire were more likely to have a university degree, to report managing financially comfortably and to own their home outright, as compared with non-respondents. Additionally, responders were less likely to be smokers, obese and reporting poor/fair SRH.

**Table 1:** A comparison of the socio-demographic and health characteristics of those who responded to the e-survey and those who did not, using data collected pre-pandemic±

	Did not respond (n = 2,985)	Responded (n = 2,469)	p-value*
Sex, men	1,311 (43.9%)	1,088 (44.1%)	0.91
Managing financially			< 0.001
Comfortably	941 (31.5%)	1,031 (41.8%)	
Doing alright	1,112 (37.3%)	896 (36.3%)	
Just about/struggling	885 (29.7%)	504 (30.4%)	
Missing	47 (1.6%)	38 (1.5%)	
Educational qualification			
No qualification/school only	1,130 (37.9%)	615 (24.9%)	< 0.001
Vocational training certificate	996 (33.4%)	614 (24.9%)	
University degree/higher	859 (28.8%)	1,240 (50.2%)	
Housing tenure			< 0.001
Owned outright	1,645 (55.1%)	1,493 (60.5%)	
Owned with mortgage	907 (30.4%)	780 (31.6%)	
Rented/rented free	384 (12.9%)	158 (6.4%)	
Missing	49 (1.6%)	38 (1.5%)	
Current smokers	309 (10.4%)	153 (6.2%)	< 0.001
Obese (BMI ≥ 30)	710 (23.8%)	505 (20.5%)	0.02
Fair/poor SRH	647 (21.7%)	335 (13.6%)	< 0.001

* p-value from Chi2 test; ± values shown are N (%)

As shown in Table 2 44% of the sample was male, mean age at completion was almost 66 years (SD = 4.3); 14% of the sample reported themselves in fair/poor SRH pre-pandemic. Just over half of the sample reported themselves financially comfortable in the pre-pandemic period, 33% was doing alright while almost 13% reported struggling financially. Just over half of the participants held a university degree. 62% of the sample owned their home outright, 32% with a mortgage and the remaining 6% were living in rented accommodation. The commonest types of employment amongst respondents to the e-survey were either professional occupations or administrative and associate professional types of jobs. The questionnaire responses to the HEAF COVID-19 survey showed that approximately half of the sample was already retired before the pandemic, while similar percentages remained working in the same place (19%) and were working from home during the pandemic (18%). About 10% stopped working during the first lockdown, 4.5% of whom were furloughed and 5.6% of whom reported that they were made redundant or decided to retire (not working – other reasons).

**Table 2:** Characteristics of the 2,344 participants included in the analyses

	N (%)
*Collected at previous follow-ups*	
Fair/poor SRH (2019)	330 (14.1)
Managing financially (2019)	
Comfortably	1,270 (54.2)
Doing alright	772 (33.0)
Just about getting by	252 (10.8)
Finding it difficult	31 (1.3)
Finding it very difficult	16 (0.7)
Educational qualification (baseline)	
No qualification/school only	581 (24.8)
Vocational training certificate	570 (24.3)
University degree/higher	1,193 (50.9)
Social class (2017-18)	
Managers, directors and senior officials	142 (12.0)
Professional occupations	305 (25.9)
Associate professional and technical occupations	173 (14.7)
Admin and secretarial occupations	213 (18.1)
Skilled trades occupations	96 (8.1)
Caring, leisure and other service occupations	87 (7.4)
Sales and customer service occupations	51 (4.3)
Process, plant and machine operatives	65 (5.5)
Elementary occupations	48 (4.1)
Housing tenure (baseline)	
Owned outright	1,433 (62.1)
Owned with a mortgage	744 (32.2)
Rented	125 (5.4)
Rented free	7 (0.3)
*Collected within the COVID-19 survey*	
Sex, men	1,042 (44.5)
Age, years mean (SD)	65.7 (4.3)
Changes in employment during the first lockdown	
Already retired pre-lockdown	1,230 (52.5)
Working in the same place	446 (19.0)
Working from home	432 (18.4)
Not working – furloughed	105 (4.5)
Not working – other reasons	131 (5.6)

*Missing values: 4 for managing financially; 35 for housing tenure; social class only available for participants in work at FU5; 9 for SRH

**Table 3:** Distribution of self-reported health outcomes during the first lockdown, overall and by categories of predictors

	Worsening of mental health N (%)	Worsening of physical health N (%)	Worsening of SRH N (%)
Overall	468 (20.8)	635 (27.4)	396 (17.0)
*SEP*			
Managing financially (2019)			
Comfortably	190 (15.5)	267 (21.3)	167 (13.2)
Doing alright	178 (24.1)	240 (31.6)	159 (20.7)
Just about/struggling	100 (35.2)	127 (43.2)	68 (22.9)
Educational qualification (baseline)			
No qualification/school only	120 (21.8)	160 (28.1)	100 (17.3)
Vocational training certificate	111 (20.4)	160 (28.3)	99 (17.5)
University degree/higher	237 (20.5)	315 (26.7)	197 (16.6)
Social class (2017–2018)			
Managers, directors and senior officials	30 (21.4)	33 (23.6)	18 (12.7)
Professional occupations	62 (20.9)	83 (27.6)	66 (21.6)
Associate professional and technical occupations	49 (28.5)	47 (27.5)	38 (22.1)
Admin and secretarial occupations	49 (23.6)	57 (26.9)	45 (21.2)
Skilled trades occupations	11 (11.8)	17 (18.1)	13 (13.7)
Caring, leisure and other service occupations	19 (22.9)	24 (28.2)	17 (19.5)
Sales and customer service occupations	10 (20.4)	22 (43.1)	13 (25.5)
Process, plant and machine operatives	9 (14.3)	20 (30.8)	8 (12.3)
Elementary occupations	9 (18.8)	14 (29.2)	7 (14.6)
Housing tenure (baseline)			
Owned outright	252 (18.3)	360 (25.5)	227 (15.9)
Owned with mortgage	171 (23.7)	210 (28.5)	133 (18.0)
Rented/rented free	33 (26.8)	54 (41.2)	31 (23.5)
Changes in employment during the first lockdown			
Already retired pre-lockdown	228 (19.8)	331 (27.3)	194 (15.8)
Working in the same place	79 (18.0)	111 (25.3)	57 (12.8)
Working from home	99 (23.1)	123 (28.7)	90 (20.9)
Not working – furloughed	26 (25.5)	28 (26.7)	21 (20.0)
Not working – other reasons	36 (27.9)	42 (32.3)	34 (26.0)

* Missing values: 92 for worsening of mental health; 30 for worsening of physical health; 9 for worsening of SRH

Table 3 reports the prevalence of self-reported health outcomes, overall and by categories of exposures. Respondents indicated that 21%, 27% and 17% of individuals reported a deterioration in their mental, physical health or SRH since the start of lockdown, respectively. The prevalence of each outcome varied significantly across categories of pre-pandemic financial status. Worsening of mental health was reported by 15% of those comfortable financially and 35% of those struggling financially, while the difference in the prevalence of worsening of physical health was more pronounced. The proportion of participants who reported a worsening of physical health was 41% among people living in rented accommodation (as opposed to 26% among those owning their homes outright). No differences were seen for the prevalence of the outcomes by educational attainment, therefore we decided not to use this variable for further analyses. Worsening of physical health was particularly prevalent among participants employed pre-pandemic in sales and customer service occupations (43.1%) (in 2017–2018), while those employed in skilled trades occupations reported the lowest prevalence of all outcomes. In terms of changes in employment status, those who stopped working in lockdown (for reasons other than being furloughed) tended to report a higher prevalence of adverse health outcomes, while participants whose job did not change reported the lowest prevalence.

**Figure 1:**
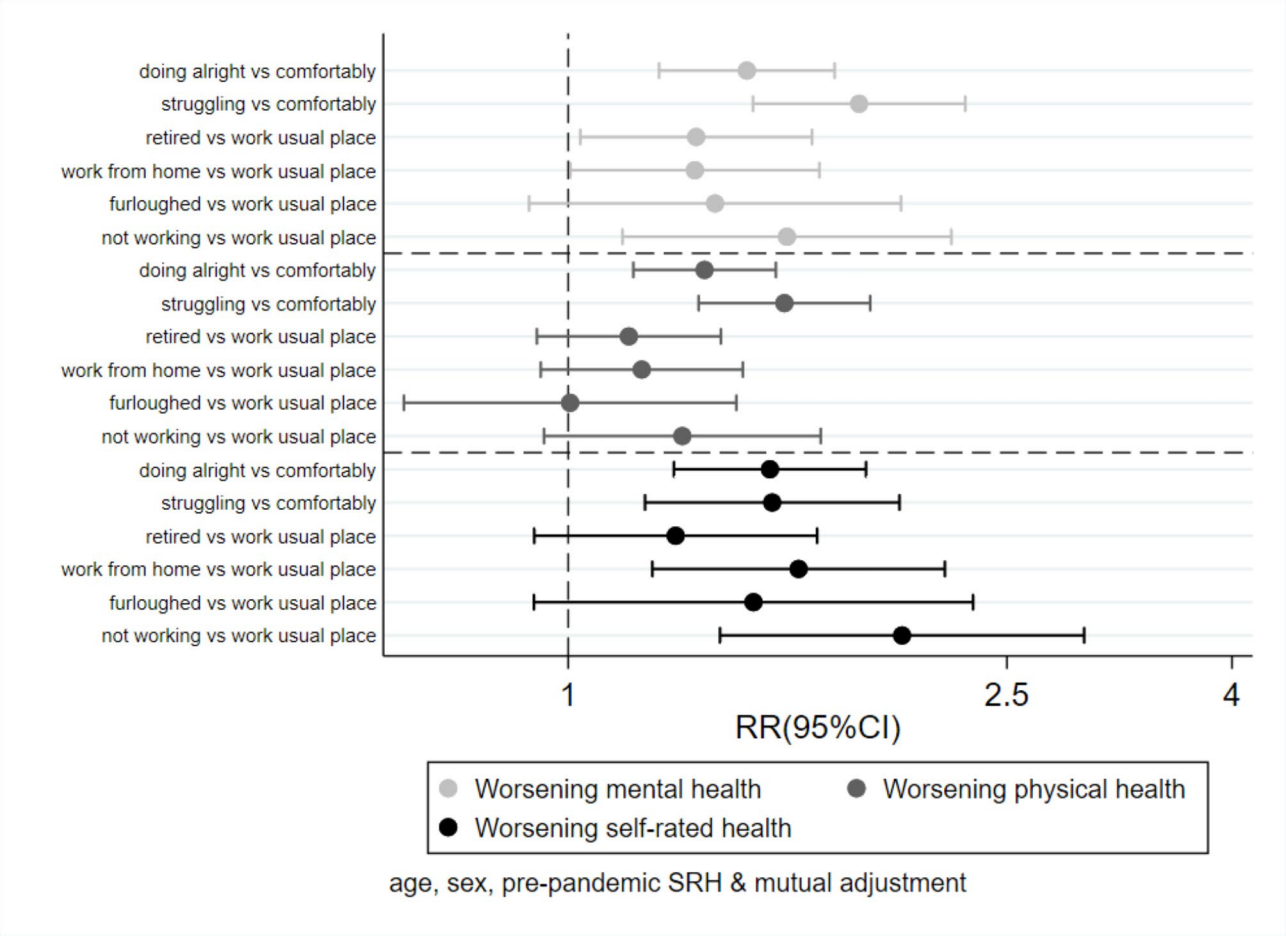
Association between changes in employment, pre-pandemic finances and changes in health

Figure 1 shows that after adjustment for age, sex, and pre-pandemic SRH, doing alright and struggling financially in the pre-pandemic period (as opposed to living comfortably) were both associated with an increased risk of worsening of mental, physical health and SRH, even after changes in employment during the pandemic were accounted for. After mutual adjustment for pre-pandemic financial status, participants working from home during lockdown and those who stopped working for any reason (as opposed to those whose employment remained unchanged) were at increased risk of reporting a worsening of mental health and worsening SRH since the beginning of the pandemic. The same trends were not seen for physical health.

**Figure 2:**
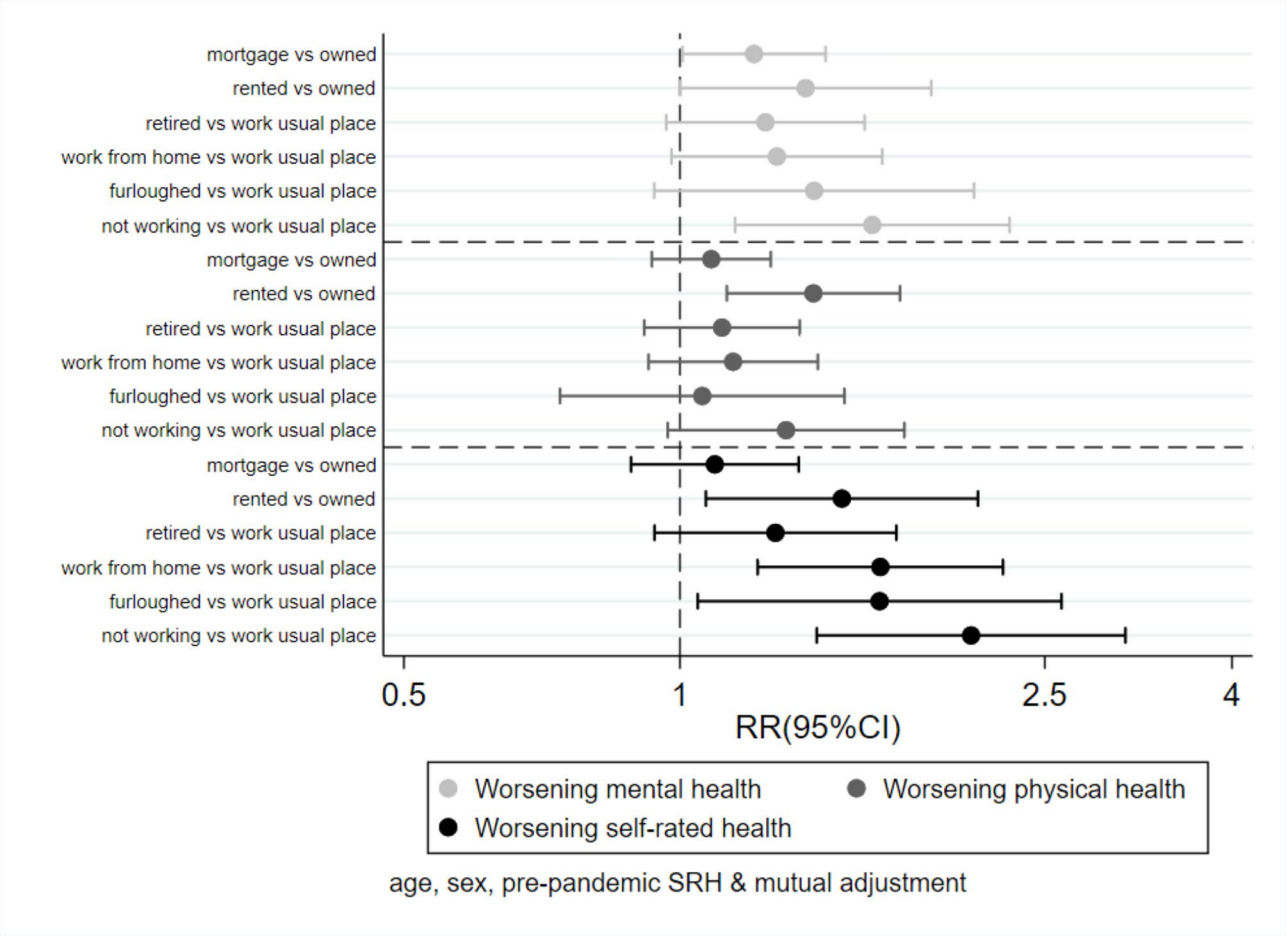
Association between changes in employment, pre-pandemic housing tenure and changes in health

Figure 2 shows the equivalent estimates where financial status was replaced by housing tenure. Independently of sex, age, pre-pandemic SRH and changes in employment, living in rental accommodation (vs. owning the home outright) was associated with an increased risk of each of the outcomes. Participants working from home (RR = 1.7, 95%CI 1.2 to 2.3), furloughed (RR = 1.7, 95%CI 1.0 to 2.6), and no longer working for other reasons (RR = 2.1, 95%CI 1.4 to 3.1) were at increased risk of worsening of SRH after adjustment for housing tenure. Participants who stopped working were also at increased risk of worsening of mental health.

**Figure 3:**
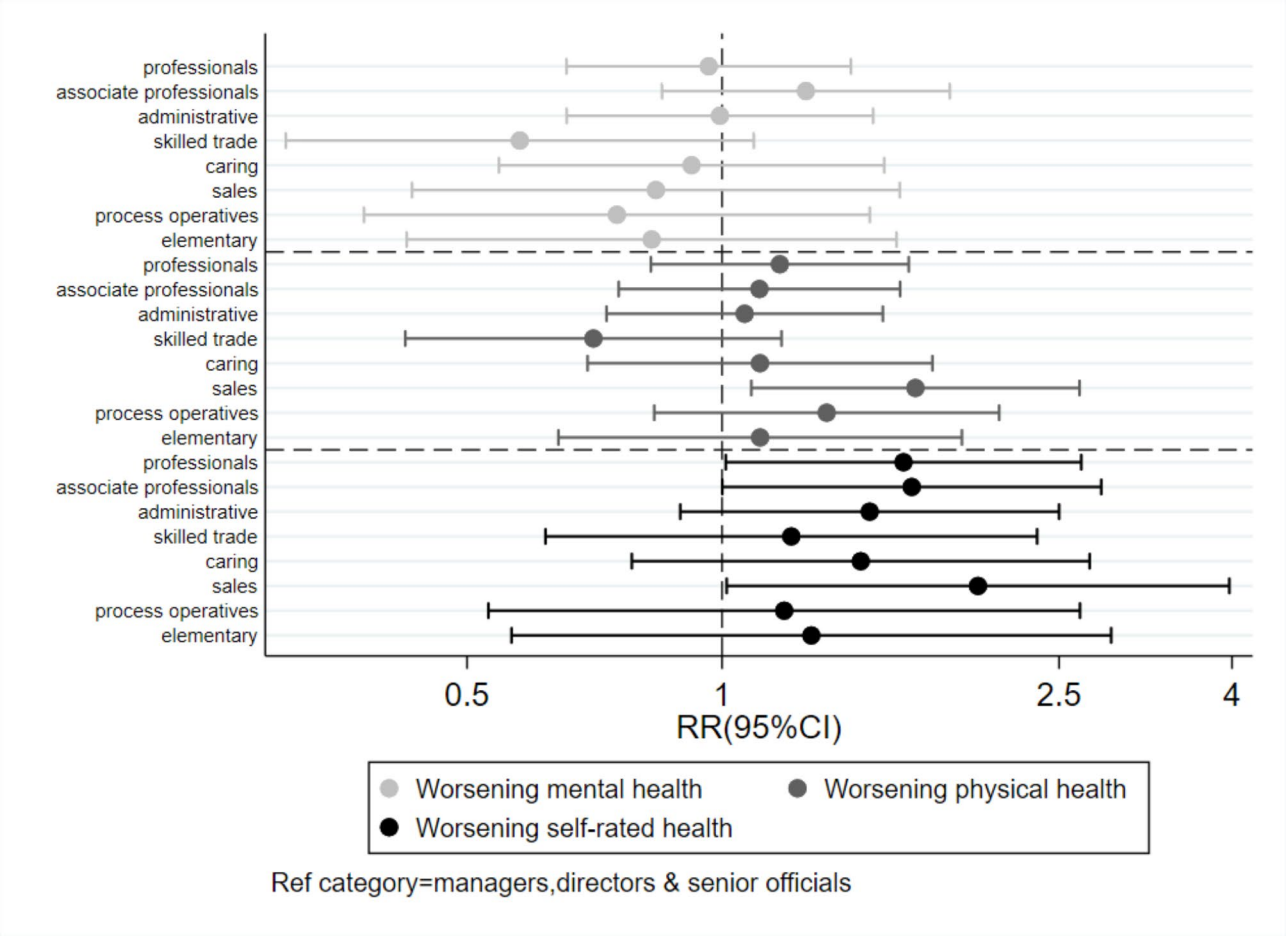
Association between pre-pandemic social class from occupation and changes in health

Finally, fully adjusted analyses showed that participants working in sales and customer service occupations were more likely to report a worsening of physical health (RR = 1.7, 95%CI 1.1 to 2.6) and a worsening of SRH (RR = 2.0, 95%CI 1.0 to 4.0) compared with those employed in managerial occupations. Working in professional occupations and associate professional occupations was also associated with an increased risk of reporting deterioration of SRH during the first lockdown (Fig. 3).

## Discussion

Our findings show that middle-aged people in England who reported themselves to be more disadvantaged financially before the pandemic as well as those who were living in rented accommodation, were at increased risk of reporting deterioration of mental health, physical health, and self-rated health since the start of lockdown. Also, having jobs in sales and customer service occupations, professional occupations and associate professional occupations was associated with worsening of SRH. These results were robust to adjustment for age, sex, pre-pandemic self-rated health, and changes to their employment status that occurred during the first lockdown. Furthermore, stopping working for any reason during lockdown was significantly associated with an increased risk of reporting deterioration in mental, physical health and SRH, while working from home in lockdown was associated with increased risk of worsening of mental health and SRH but not physical health.

These findings need to be considered alongside some limitations. Firstly, both health outcomes and predictors were self-reported, but the predictors were reported pre-pandemic in 2019 by a cohort of people recruited to a longitudinal study of health, ageing and retirement. Self-rated health has frequently been shown to be an excellent proxy for other markers of physical health and mortality [21, 22]. Secondly, information about housing tenure was collected at the time of the baseline questionnaire (in 2013–2014) and social class was derived from occupation and industry as reported by participants pre-pandemic (2017–2018), meaning that we could not be certain they corresponded to the participant’s status in the period just before lockdown. Additionally, because of small numbers in some categories of changes in employment (i.e. furloughed) we cannot rule out the possibility that there might have been an association with the outcomes that we were underpowered to detect. Finally, these analyses suffer from responder bias as participants who returned a usable questionnaire reported better SEP and health status at baseline, as compared with the remaining consenting participants. Given that respondents to this survey were generally better off (likely because of better access to e-mail), it may well be therefore that our results reflect an under-estimate of the full effects of the pandemic on those worst off in society.

The main strengths of this study lie in its longitudinal design which enabled us to investigate the effect of pre-pandemic factors collected well before anyone had heard of COVID-19 and changes in health as perceived by participants during the pandemic.

Our findings of an association between job loss and worsening of health (mainly mental health) are consistent with what was recognised in the pre-pandemic literature in which unemployment was associated with poor mental health. [23] Some post-pandemic studies have reported similar findings, although there has been a lack of consistency about which type of health outcomes have been assessed. For example, one cross-sectional study of 2,300 people aged 18 + in the US found that those who lost a job because of the pandemic reported more symptoms of depression, anxiety, and stress, and lower levels of positive mental health, as compared with those with an unchanged job position [8]. Griffith et al. analysed a sample of 2,600 Australians aged 18 + and found that those who lost their job during lockdown as well as those not working for other reasons (furloughed or took leave) were at increased risk of high levels of psychological distress, poor mental health, and poor physical health. Additionally, these researchers also found that these associations were more pronounced in the subgroup experiencing financial hardship. [9] Likewise, a survey of Australians conducted early after COVID-19 restrictions found that those who had lost their jobs since the pandemic were more likely to report clinically significant symptoms of depression or anxiety compared to those whose job was not affected. [24] Comparing self-employed people whose business had not been affected by lockdown with people who were either made redundant, became unemployed or who reduced their working hours during lockdown, Chandola and colleagues found that there were increased odds of common mental disorders amongst those whose job status or hours changed. In the same analysis however increased odds of common mental disorders were not found for those who were furloughed. [7] Finally, a longitudinal study conducted on a South-African adult population showed that those who retained employment during the COVID-19 lockdown reported significantly lower depression scores than adults who lost employment or who were furloughed. Such associations were robust to adjustment for pre-pandemic socio-economic position. [10] Taken together therefore, it is clear that health, particularly mental health, had been adversely affected by the pandemic but that maintenance of employment was an important factor in mitigation. Given that the pandemic was associated with many life restrictions and considerable uncertainty about risks to individuals and their families, it is interesting to see that sustained employment was able to ameliorate these anxieties to some extent. This would seem to emphasise the message that good work is good for health.

It is fascinating that our data suggested a differential effect of the lockdown on people working in professional services, retail, professional occupations, and associate professional occupations. We hypothesise that people in these types of occupations were either “essential” workers who worked under higher pressures during the pandemic (e.g. in retail sector due to shortage of goods) or were those in management roles in businesses that found themselves having to make decisions about operating their businesses safely, furloughing employees, fulfilling contracts, leasing premises etc. Mental health status of healthcare workers has been the focus of several papers who have identified these workers at increased risk of reporting anxiety, depression, and sleep disturbances. [25–27] Other papers have explored the mental health impact of the pandemic on essential workers employed in retail, food service or hospitality [28] [29] and have found them to be at increased risk of reporting moderate levels of anxiety compared with non-essential workers.

Interestingly, although with wide confidence intervals, we did not find that working in the “caring” sector during the first lockdown was associated with an increased risk of adverse health outcomes. This may reflect that this older worker sample of people working in the caring professions were perhaps less exposed to the “front line”, at least at the time of the survey, or that those still working in this sector at mean age 66 years were particularly resilient “healthy workers”. That being in the skilled trades was associated with a reduced risk of deteriorating health is also interesting. It is possible that this may reflect that workers in these jobs retained both employment and some autonomy throughout the lockdown as they would have been “essential” workers. It will be important to know, going forwards, to what extent the adverse effects are reversed as economies start to recover and indeed whether older workers are able to return successfully to the workforce.

In our study, home working was associated with reporting a deterioration in health. The findings from other studies are more variable, however. Chandola et al. found that working from home every day since lockdown, as compared with never working from home, was associated with higher odds of common mental health disorders. [7] Similarly, whilst an analysis of the UK Household Longitudinal Study observed a decrease in mental well-being score during the pandemic for all adults sampled, they found a greater decrease amongst those who reported switching to home working constantly (as opposed to those who never worked at home). [30] In contrast however, a cross-sectional study of American adults found that switching to working from home was not associated with worsening of any of: depression; anxiety; stress; or positive mental health. [8] A rapid review which included papers published before and after lockdown [31] found that the evidence about the impact of home-working on health (physical and mental) was conflicting: some studies found that home-working increased wellbeing while others found that it increased stress. One of the reasons for the different findings might be the use of different case definitions for home working. In the current study, we employed an inclusive definition which asked anybody who had done any home working during lockdown to respond positively but we did not ask if they had done any home working pre-pandemic. Other studies have found that health has been more affected for people who transitioned to home working during the pandemic as compared with those who already had experience of home working pre-pandemic. Importantly, under normal circumstances, it could be that people work from home for a wide range of different reasons: to accommodate caring responsibilities; because of personal health issues; or because of the nature of their employment. It may be that home working is good for health when it is a personal choice and/or is well-supported by the employer. The unique aspect of home working in the pandemic was the massive scale of it and that it occurred without warning or time for adjustment and was often required to be performed whilst also home schooling and amidst widespread fear for safety. In this context too, it is perhaps not surprising that health effects are inconsistent. Some people will have benefitted from a loss of their commute, gained more time for leisure-time physical activity, and enjoyed a better diet and work/life balance, whilst others will have struggled to work with an inadequate workstation using poor technology with children trying to share their bandwidth to undertake their home studies.

Although not surprising, our findings that pre-pandemic socio-economic status was associated with health outcomes during the COVID-19 pandemic is important. It is well-known that there are social inequalities in health including mental health [32] and health in later life.[18] This suggests that not only were mortality and morbidity greater amongst those from poorer socio-economic backgrounds [33] but also that the pandemic has differentially affected mental health, physical health and self-rated health. It will be important to see whether these effects are reversed as economies recover or whether more lasting effects are felt differentially. In particular, if people who do not own their own home or are struggling to manage financially are too unwell to re-join the labour market, then the pandemic will have exacerbated health inequality and, given the strong association between SRH and mortality and morbidity [21, 22], potentially threatened the post-pandemic quality and quantity of lives amongst the poorest in society.

## Conclusion

In this paper we have demonstrated evidence of differential effects of the lockdown on the health of older workers, depending upon their pre-pandemic financial circumstances and changes in employment status that occurred during the pandemic. More research is needed to explore whether these short-term effects of the pandemic are sustained in the long term.

## Data Availability

The datasets used for this analysis are available on reasonable request from the MRC Versus Arthritis Centre for Musculoskeletal Health and Work by contacting Dr. Catherine Linaker: chl@mrc.soton.ac.uk.
